# Impacts of Small RNAs and Their Chaperones on Bacterial Pathogenicity

**DOI:** 10.3389/fcimb.2021.604511

**Published:** 2021-07-12

**Authors:** Louise Djapgne, Amanda G. Oglesby

**Affiliations:** ^1^ Department of Chemistry, Georgetown College, Washington, DC, United States; ^2^ Department of Pharmaceutical Sciences, University of Maryland School of Pharmacy, Baltimore, MD, United States; ^3^ Department of Microbiology and Immunology, School of Medicine, University of Maryland, Baltimore, MD, United States

**Keywords:** sRNA, Hfq, ProQ, FinO, RsmA, Crc

## Abstract

Bacterial small RNAs (sRNAs) are critical post-transcriptional regulators that exert broad effects on cell physiology. One class of sRNAs, referred to as *trans*-acting sRNAs, base-pairs with mRNAs to cause changes in their stability or translation. Another class of sRNAs sequesters RNA-binding proteins that in turn modulate mRNA expression. RNA chaperones play key roles in these regulatory events by promoting base-pairing of sRNAs to mRNAs, increasing the stability of sRNAs, inducing conformational changes on mRNA targets upon binding, or by titrating sRNAs away from their primary targets. In pathogenic bacteria, sRNAs and their chaperones exert broad impacts on both cell physiology and virulence, highlighting the central role of these systems in pathogenesis. This review provides an overview of the growing number and roles of these chaperone proteins in sRNA regulation, highlighting how these proteins contribute to bacterial pathogenesis.

## Introduction

The World Health Organization (WHO) published on its website in 2017 that infectious diseases are among the leading causes of death in the world. The ultimate challenge to manage the impact of infectious diseases is to better understand processes guiding microbial pathogenesis. An organism’s ability to respond to environmental stresses is critical for survival, and bacterial pathogens have long been known to mediate gene expression changes in response to host-specific cues including antibiotics, carbon source, temperature, pH, reactive oxygen species, and metal nutrients. Through accurate and timely regulation of virulence gene expression, bacteria not only efficiently evade the host immune system, but also save energy and avoid the potential toxicity of excess nutrients.

Small bacterial regulatory RNAs (sRNAs) have gained immense appreciation over the past two decades for their roles in mediating post-transcriptional gene regulation of numerous physiological and virulence-related processes in bacteria by synchronizing complex networks of stress adaptation [recently reviewed by (Chakravarty and Masse, 2019)]. In many cases, sRNAs are dependent upon so-called “RNA chaperones”, which function by binding to secondary and tertiary structures of RNA molecules and inducing structural changes that allow for their function [recently reviewed by (Quendera et al., 2020)]. The purpose of this review is to highlight the increasingly appreciated role that RNA chaperones play in mediating sRNA-dependent regulation of virulence gene expression in bacterial pathogens. We begin by providing an overview of bacterial sRNA gene regulation mechanisms, including how RNA binding proteins contribute to these regulatory pathways. We next highlight four major physiological processes – outer membrane transport, biofilm formation, iron homeostasis, and quorum sensing – that are affected by Hfq-dependent sRNAs, and we discuss how Hfq regulation of individual processes may contribute to successful infection. Lastly, we discuss the growing appreciation of additional RNA chaperones, and we pose questions for the field to consider to better understand the impact of sRNA regulation on bacterial pathogenesis.

## Bacterial sRNA-Mediated Gene Regulation

Bacterial sRNAs play a major role in post-transcriptional regulation of bacterial gene expression, and RNA chaperones are critical for the function of many of these sRNAs. Work in numerous model organisms has shed light on the biochemical and molecular bases of both how sRNAs function and the role of RNA chaperones in their activity. In general, sRNAs have been classified as either *trans*-acting sRNAs, which directly pair with mRNAs, or as Csr/Rsm-type sRNAs, which sequester post-transcriptional regulatory proteins away from target mRNAs. Below we provide an overview of each type of sRNA and how RNA chaperones affect their function. We also discuss the Crc protein, which was originally thought to function as an RNA-binding protein that could be sequestered by the CrcZ sRNA, but it is now understood to work in concert with Hfq to modulate post-transcriptional regulation.

### *Trans*-Acting sRNAs

*Trans*-acting bacterial sRNAs function *via* limited base pairing with mRNA targets, resulting in either negative or positive effects on gene expression. Negative regulation often occurs when sRNA pairing occurs at or near the Shine Dalgarno (SD) and/or translational start site of the mRNA, precluding access to the ribosome and leading to increased susceptibility to RNases (Aiba, 2007). *Trans*-acting sRNAs can also exert positive effects on mRNA stability and translation, most often by inducing conformational changes in the untranslated region to promote ribosomal binding (Prevost et al., 2007; Soper et al., 2010). Several proteins contribute to bacterial sRNA stability and function, most notably the Hfq RNA-binding protein encoded by many bacterial species (Brown and Elliott, 1996; Christiansen et al., 2004; Arluison et al., 2007; Argaman et al., 2012). However, not all sRNAs rely on Hfq for their stability or function (Bohn et al., 2007; Deng et al., 2012), and some sRNA-expressing bacteria do not encode an obvious *hfq* homolog. Recent work shows that a second RNA chaperone, ProQ, contributes to regulation by a distinct class of sRNAs in several Gram-negative bacteria, including *E. coli*, *Salmonella*, and *Legionella* (Olejniczak and Storz, 2017). While these two types of RNA chaperones mediate a range of sRNA regulation in Gram-negative bacteria, they are not required for the function of many other sRNAs. Moreover, while Hfq is encoded by many Gram-positive bacteria, sRNA regulation in these organisms largely occurs independently of Hfq. Further work is needed to determine if Hfq- and ProQ-independent sRNAs function absent a protein chaperone, or if novel classes of RNA chaperones modulate the activity of these sRNAs.

### Csr/Rsm-Type sRNAs

Bacterial sRNAs can also regulate gene expression by sequestering RNA-binding proteins, which is exemplified by the CsrA/RsmA family. In *E. coli*, CsrA (carbon storage regulatory protein) regulates central carbon flux as well as biofilm formation and motility (Weilbacher et al., 2003). CsrA alters gene expression by binding to GGA motifs within loops of the mRNA structures, thereby changing the stability of the mRNA targets and affecting translation initiation (Babitzke and Romeo, 2007). CsrA can alternatively be sequestered by the sRNAs CsrB and CsrC, which bind to CsrA with high affinity (Babitzke and Romeo, 2007; Storz et al., 2011). CsrB contains 22 CsrA binding sites (GGA motifs) and is capable of sequestering about 9 CsrA dimers, while the CsrC sRNA contains only 9 CsrA binding motifs (Weilbacher et al., 2003). The levels of the Csr sRNAs are controlled in part by the CsrD protein, which targets the sRNAs for degradation, while Csr sRNA stability is not affected by either the CsrA or Hfq proteins [reviewed by (Vakulskas et al., 2015)]. The Csr system has been identified in numerous bacterial species, although the number of Csr sRNAs and CsrA-like proteins expressed by different species varies dramatically (Lenz et al., 2005; Vakulskas et al., 2015).

*Pseudomonas* species and the plant pathogen *Erwinia caratovora* possess homologs of the Csr system that were originally named Rsm (for repressor of secondary metabolites). Similar to the Csr systems, Rsm systems are comprised of one or more sRNAs (RsmX, RsmY, RsmZ, RsmV) and one or more CsrA-like proteins (RsmA, RsmB, RsmF, RsmE, RsmW) (Storz et al., 2011; Romero et al., 2016; Janssen et al., 2018). Studies in *E. caratovora* initially described and named the RsmA protein for its functions as a global regulator of extracellular enzymes, quorum sensing, and pathogenesis (Chatterjee et al., 1995; Babitzke and Romeo, 2007). A single RsmB sRNA in this species was revealed to contain multiple RsmA GGA binding motifs on its stem loops, and its ability to titrate RsmA from target genes using these GGA motifs was established. This system functions similarly in *Pseudomonas fluorescens* and *Pseudomonas aeruginosa*, but with multiple Rsm sRNAs and RsmA-type proteins (Storz et al., 2011; Romero et al., 2016; Janssen et al., 2018). The Rsm systems in the Pseudomonads lack a CsrD-like protein to control stability of the sRNAs. However, in both *P. fluorescens* and *E. caratovora*, the RsmA-type proteins protect the Rsm sRNAs from cellular nucleases (Chatterjee et al., 2002; Reimmann et al., 2005), and in *P. aeruginosa* Hfq contributes to the stability of RsmY (Sonnleitner et al., 2006). The difference in how the stabilities of the Csr and Rsm sRNAs are modulated is likely reflective of the different physiologies of the organisms by which they are produced. Degradation of the Csr sRNAs by CsrD is responsive to the glucose-specific phosphotransferase system (PTS), which senses the presence of glucose to mediate carbon catabolite repression (CCR) in enteric bacteria (Leng et al., 2016). The molecular mechanisms guiding CCR in the Pseudomonads are vastly different than that of the enterics, in large part due to the preference of the Pseudomonads for organic acids over sugars (Rojo, 2010). Thus, regulatory control of the Csr and Rsm systems likely evolved in response to specific carbon sources that were available in their respective habitats.

### Hfq-Crc Regulation of Catabolite Repression

CCR in the Pseudomonads is mediated at the post-transcriptional level through the catabolite repression control (Crc) protein and the CrcZ sRNA (Moreno et al., 2007). The original model for Crc function posited that Crc bound mRNAs involved in carbon utilization and modulated their translation and/or stability (Moreno et al., 2007). However, more recent studies have shown that Crc has no RNA binding activity (Milojevic et al., 2013) and suggest that Crc instead promotes binding of Hfq to target mRNAs (Sonnleitner and Blasi, 2014; Moreno et al., 2015; Pei et al., 2019). Activity of the Crc-Hfq complex is countered by the CrcZ sRNA, whose expression increases when preferred carbon sources are depleted (Nishijyo et al., 2001). CrcZ was originally shown to have a high binding affinity for Crc and was therefore thought to sequester this protein away from mRNA targets (Elisabeth Sonnleitner and Haas, 2009). However, with the finding that Crc itself does not bind to RNA, this model too has been re-evaluated, with more recent work showing that CrcZ binds to Hfq and titrates it away from its mRNA targets (Sonnleitner and Blasi, 2014). Notably, CrcZ can also titrate Hfq from other sRNAs, including the iron-responsive PrrF sRNAs, to reduce their stability and activity (Sonnleitner et al., 2017). This series of studies demonstrates the interconnectivity of sRNA regulatory networks, a consequence of their dependency on RNA chaperones.

## Impacts of Hfq on sRNA Regulation

**Figure 1:**
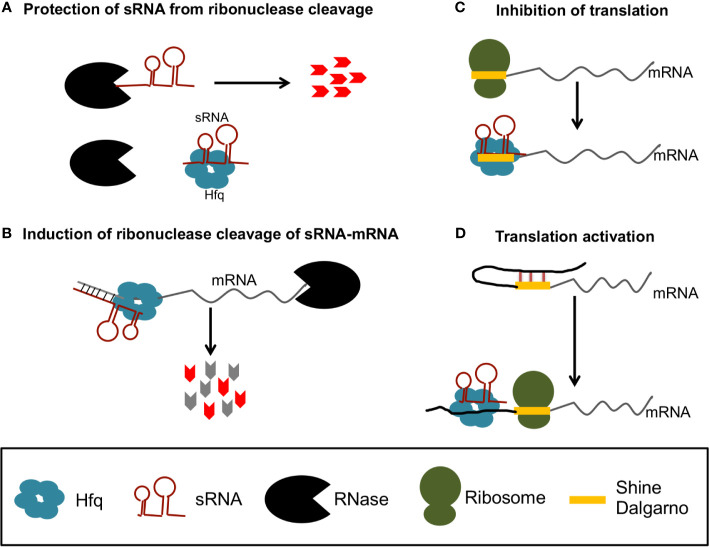
Gene regulation by sRNA and Hfq in bacteria. Hfq can protect sRNAs from ribonuclease cleavage **(A)** or recruit RNases to degrade of sRNA-mRNA complexes **(B)**. Hfq can also promote sRNA binding that precludes access of the ribosome to the Shine Dalgarno (SD) to inhibit translation **(C)**, or promote sRNA binding that releases inhibitory structures to increase translation **(D)**.

The best studied sRNA chaperone protein is host factor Q (Hfq) [reviewed by (Updegrove et al., 2016)], which was originally identified as a required host factor for replication of the RNA phage Qβ (Franze de Fernandez et al., 1968). Hfq’s role in sRNA regulation has been primarily described in *E. coli*, where Hfq can bind and stabilize sRNAs, mRNAs, or sRNA-mRNA complexes (**Figure 1A** **)** (Soper et al., 2010), or can alternatively target mRNAs and mRNA-sRNA complexes for degradation by recruiting RNase E (**Figure 1B**) (Mohanty et al., 2004; Ikeda et al., 2011). Alternatively, Hfq can alter mRNA translation efficiency when bound to mRNA-sRNA complexes, either by precluding ribosome access to the Shine Dalgarno (SD) (**Figure 1C**) or by altering mRNA structures to increase access to the SD (**Figure 1D**).

### Structural Elements of Hfq-Dependent sRNA Regulation

**Figure 2:**
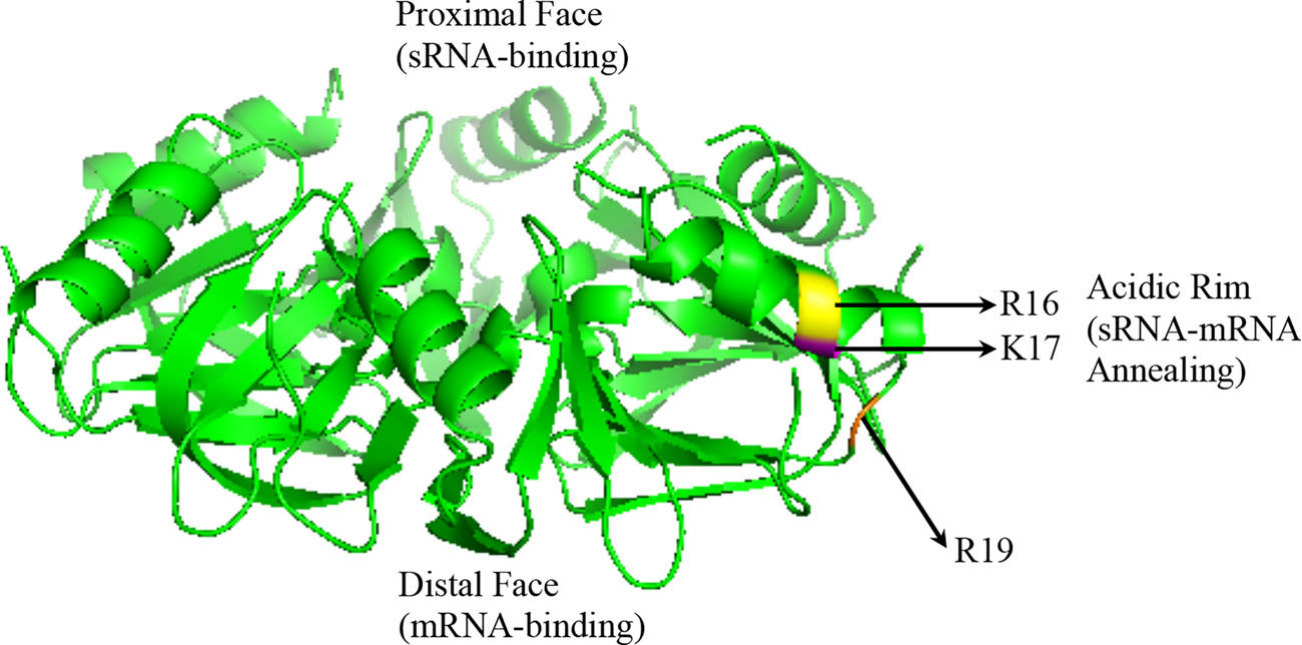
Crystal structure of the *P. aeruginosa* Hfq protein adapted from 1U1S.PDB: its proximal face, its distal face, and its rim/lateral face indicated. Also indicated in yellow (R16), orange (K16), and magenta (R19) are the acidic amino acid residues on the rim (Nikulin et al., 2005).

Hfq recognizes unstructured regions of sRNAs and/or mRNAs in a semi-specific manner (Hopkins et al., 2011; Beisel et al., 2012; Peng et al., 2014). The structure of all Hfq proteins crystallized to date reveals a hexameric ring (**Figure 2**) (Someya et al., 2012; Horstmann et al., 2012; Santiago-Frangos et al., 2019). U-rich regions of most *E. coli* sRNAs, termed Class I sRNAs, are recognized by the proximal face of Hfq ring, while Hfq’s distal face recognizes A-rich regions of mRNA (Hopkins et al., 2011; Zheng et al., 2016). The lateral or “rim” face, which bears a positive charge due to arginine and/or lysine residues, mediates sRNA-mRNA annealing (Panja et al., 2013). A second and smaller set of *E. coli* sRNAs, termed Class II, bind to both the proximal and distal faces of Hfq, while the mRNA interacts with the Hfq rim (Zhang et al., 2013; Schu et al., 2015). This latter Class II of sRNAs appears to be more stable, due at least in part to A-rich motifs that allow for binding to Hfq’s distal face (Lapouge et al., 2008; Schu et al., 2015). The stability and binding mode of Class II sRNAs may also allow these molecules to more efficiently compete with Hfq (Kwiatkowska et al., 2018).

### Hfq Increases sRNA-mRNA Annealing Rates

Studies of Hfq established that the chaperone protein promotes annealing between some sRNAs-mRNA pairs, in many but not all bacterial species (Soper and Woodson, 2008; Peng et al., 2014). In *E. coli*, Hfq uses a positively-charged arginine patch on its lateral face to initiate the formation sRNA-mRNA pair (Panja et al., 2013; Zheng et al., 2016). Therefore, Hfq was suggested to facilitate base pairing between sRNA and mRNA by increasing annealing rates (Fender et al., 2010; Hopkins et al., 2011; Hwang et al., 2011). One well-studied example in *E. coli* is the ability of Hfq to accelerate the rate of annealing between DsrA sRNA and the *rpoS* mRNA. Either the DsrA sRNA or *rpoS* mRNA could bind to Hfq to initiate the formation of the ternary complex; the interaction of Hfq with either one of these RNAs induced an unwinding of the initiating RNA’s structure to facilitate base pairing with the other RNA (Fender et al., 2010; Hopkins et al., 2011; Hwang et al., 2011). Another set of studies demonstrated that instead of simultaneously binding, OmrA/OmrB sRNAs and their *dcgM* mRNA target to facilitate annealing, *E. coli* Hfq binds the *dcgM* mRNA and promotes unfolding of two stem-loops that inhibit the sRNAs access to the ribosome binding site (Hoekzema et al., 2019). Hfq thus destabilizes the mRNA secondary structure to allow access to the sRNA, which in turn blocks the ribosome-binding site and therefore ablates translation.

Mechanistic studies of Hfq’s contribution to sRNA regulation in species other than *E. coli* are more limited. Recent studies using fluorescent molecular beacon RNAs showed differential annealing activities of the Hfq proteins in *P. aeruginosa* and *E. coli* when compared with those encoded by Gram-positive bacteria. *E. coli* Hfq, which possesses three arginine residues on its rim (R16, R17, R19), promoted annealing of the fluorescent beacon at a rate 100-fold higher than without Hfq present, while *P. aeruginosa* Hfq, which possesses a single R->K substitution (R16, K17, R19) only increased the rate of annealing by 10-fold. *L. monocytogenes* Hfq, which possesses two R->K substitutions on its rim (R16, K17, K19) increased annealing by 3-fold, while both *B. subtilis*, which contains only one arginine (R16, K17, N19), and *S. aureus*, which lacks an arginine patch entirely (K16, A17, Q19), were unable to initiate sRNA-mRNA annealing (Zheng et al., 2016). Additional studies of *P. aeruginosa* Hfq with a native sRNA-mRNA binding pair showed that Hfq similarly increases the rate of sRNA annealing (Sonnleitner et al., 2017; Djapgne et al., 2018). Of note, the rims of the Hfq proteins in non-pathogenic *Pseudomonas* species possess three lysine residues, suggesting a reduced ability of these Hfq proteins to facilitate sRNA-mRNA annealing. Thus, it remains unclear how broadly conserved the annealing function of Hfq is, underlining how much of this protein’s function is independent of sRNA regulation [reviewed in (Dos Santos et al., 2019)].

## Impacts of Hfq-sRNA Regulation on Bacterial Cell Physiology and Virulence

To cause infection, bacterial pathogens must be able to effectively colonize their host, acquire nutrients that are often sequestered, and evade the activities of the immune system. The timing of expression of genes that mediate these functions is directed by a complex regulatory cascade that responds to rapidly changing environmental cues. Bacterial sRNAs are critical regulators of these pathways, as they allow for bacteria to rapidly respond to changes in the environment, including those that signal nutritional requirements and evasion of host immune factors. Hfq similarly plays a critical role in many of these processes, in some cases through its modulation of sRNA function. However, due to Hfq’s pleiotropic roles in regulating gene expression, *via* both sRNA-dependent and -independent mechanisms, teasing out these functions has proven to be an immensely complicated field of study. Below we highlight four specific virulence-related processes that are responsive to both Hfq and sRNA regulation, and we describe the current knowledge of how these Hfq-dependent processes intersect to affect pathogenesis.

### Hfq-sRNA Regulation of Iron Homeostasis

Iron is a critical cofactor for nearly all-living organisms, and bacterial sRNAs are well-characterized as major mediators of bacterial iron homeostasis (Oglesby-Sherrouse and Murphy, 2013). The iron responsive RyhB sRNA was first identified in *E. coli*, and the gene from which it was transcribed was highly conserved amongst numerous enteric pathogens, including pathogenic *E. coli* strains, and *Shigella*, *Salmonella*, *Yersinia*, and *Vibrio* species (Masse and Gottesman, 2002). The RyhB sRNAs from all enteric organisms that have been studied are expressed upon iron starvation and repress the expression of iron-containing proteins to spare iron when it is limiting, and in pathogenic enterics RyhB has also been linked to virulence (Davis et al., 2005; Mey et al., 2005; Oglesby et al., 2005; Broach et al., 2012; Deng et al., 2012; Leclerc et al., 2013; Porcheron et al., 2014). Hfq is required for RyhB stability in *E. coli* K12 (Masse et al., 2003), and is similarly important for RyhB function in many, but not all enteric pathogens. In uropathogenic *E. coli* (UPEC), RyhB positively regulates siderophore biosynthesis through the regulation of two separate genes - *shiA* and *iucD* – and the siderophore defect correlated with decreased colonization in a murine model of urinary tract infection (Porcheron et al., 2014). Because of the close relation to *E. coli* K12, UPEC RyhB is presumably dependent on Hfq, though this has not been definitively shown. *Yersinia pestis* and *Yersinia pseudotuberculosis* also produce two RyhB homologs – RyhB1 and RyhB2 – both of which are expressed during mammalian infection (Deng et al., 2012). RyhB1 and RyhB2 sRNAs have distinct requirements for Hfq, with RyhB2 showing more inherent stability in the absence of *hfq* (Deng et al., 2012), thus RyhB function in *Yersinia* species may be retained in the absence of Hfq. *V. cholerae* RyhB, which interacts with the Hfq protein *in vivo* (Davis et al., 2005), also regulates multiple virulence related phenotypes, including motility, biofilm formation, but it is not required for intestinal colonization in a murine model (Davis et al., 2005; Mey et al., 2005).

Subsequent to the identification of RyhB, the iron-responsive PrrF (Pseudomonas RNAs responsive to Fe) sRNAs, were identified in *P. aeruginosa*. While the PrrF sRNAs show no sequence homology to RyhB, they mediate many of the same functions in promoting iron homeostasis (Wilderman et al., 2004). The PrrF sRNAs are also required for biofilm formation in the presence of antibiotics, and they are essential for colonization and virulence in an acute murine lung infection model (Reinhart et al., 2015; Reinhart et al., 2017). The PrrF sRNAs rely on the chaperone protein Hfq interaction with at least one of their target mRNAs, *antR* (Sonnleitner et al., 2017; Djapgne et al., 2018). PrrF repression of *antR* spares anthranilate for synthesis of a family of small, secreted molecules termed 2-akyl-4(1*H*)-quinolones (Djapgne et al., 2018), which mediate a variety of toxic effects against mammalian and microbial cells. More recent work using label-free proteomics discovered that PrrF positively affects levels of proteins for twitching motility and T6SS (Nelson et al., 2019; Brewer et al., 2020). Thus, Hfq-dependent PrrF regulation likely exerts pleiotropic effects on cell physiology and virulence gene expression, making it likely that the PrrF requirement in virulence is multifactorial. Numerous additional orthologs of RyhB have now been identified in both Gram-positive and Gram-negative bacterial species, with varying dependencies on Hfq. Like PrrF, these orthologs share little to no sequence similarities to RyhB (or PrrF), but they exert similar effects on gene expression and iron homeostasis (Mellin et al., 2007; Ducey et al., 2009; Metruccio et al., 2009; Jackson et al., 2013; Santana et al., 2014; Pannekoek et al., 2017; Sass and Coenye, 2020). However, the specific impacts of iron-responsive sRNAs and Hfq on virulence of these organisms has yet to be fully elucidated.

### Hfq-sRNA Regulation of Outer Membrane Proteins in Enteric Bacteria

Gram-negative bacteria contain numerous outer membranes proteins (OMPs) that are essential for bacterial nutrient and solute transport and therefore are critical to bacterial survival. OMPs serve to scavenge nutrients from the environment, and in pathogenic bacteria further function to evade the host defense mechanisms as well as mediate cell adhesion and signaling (Rollauer et al., 2015). The appropriate regulation of OMP biogenesis is critical for survival, and dysregulation of OMPs can lead to membrane perturbations that are sensed by the sigma E (σ^E^) regulatory protein (Ding et al., 2004; Figueroa-Bossi et al., 2006; Vogt and Raivio, 2014). Amongst the σ^E^ regulon in enteric bacteria are multiple Hfq-dependent sRNAs, including MicA, RybB, CyaR, and MicL (Johansen et al., 2006; Johansen et al., 2008; Guo et al., 2014). In response to envelope stress these sRNAs bind to and destabilize mRNAs encoding OMPs including LamB and OmpA [by the MicA sRNA (Bossi and Figueroa-Bossi, 2007)], OmpX [by the CyaR sRNA (Papenfort et al., 2008)], OmpC and OmpD [by the RybB sRNA (Johansen et al., 2006; Vogel and Papenfort, 2006)], and Lpp [by the MicL sRNA (Guo et al., 2014)]. Hfq therefore plays an integral role in membrane composition in enteric pathogens, and deletion of *hfq* in multiple enteric bacteria leads to membrane perturbations and activation of the σ^E^ stress response (Figueroa-Bossi et al., 2006; Johansen et al., 2006; Vogel and Papenfort, 2006; Guisbert et al., 2007; Guo et al., 2014; Vogt and Raivio, 2014; Klein and Raina, 2017).

### Hfq-sRNA Regulation of Biofilm Formation

Biofilms represent a major mechanism for bacterial survival during chronic infections (Costerton and Stewart, 2001; Brady et al., 2008; Hoiby et al., 2011). Owing in part to its role in membrane composition, Hfq affects biofilm formation in multiple enteric pathogens. Salmonella *hfq* mutants show defects in biofilm formation and maturation (Kint et al., 2010; Monteiro et al., 2012), phenotypes that have been attributed to multiple sRNA regulatory pathways. These include the above cited Hfq-dependent sRNAs that affect OMP expression, as well as the Hfq-dependent ArcZ, RprA, OxyS, and DsrA sRNAs that coordinate expression of the stationary phase sigma factor RpoS [reviewed in (Van Puyvelde et al., 2013)]. In *P. aeruginosa* the Rsm system controls the switch between chronic and acute virulence phenotypes *via* the modulation of the RNA-binding protein RsmA. As mentioned above, when RsmY and RsmZ small RNAs are expressed, they inhibit the activity of RsmA protein by sequestering the latter away from its mRNA targets (Storz et al., 2011; Romero et al., 2016; Janssen et al., 2018). RsmA is essential for the activation of type 3-secretion system (T3SS), which is a noted marker of acute infection; sequestration of RsmA results in the activation of type 6 secretion system (T6SS) and biofilm formation (T6SS), both recognized as a hallmarks of chronic infection (Lapouge et al., 2008). Consequently, in *P. aeruginosa*, the Rsm system influences organism pathogenicity and patient disease state by governing the transition from acute to chronic infection (Sonnleitner et al., 2006). Biofilm formation is further controlled in *P. aeruginosa* by the chaperone protein Hfq, which mediates the expression of RsmA protein in this network and thus indirectly controls the formation of biofilms (Irie et al., 2020).

### Hfq and sRNA Regulation of Quorum Sensing

Hfq-dependent sRNAs have also been shown to regulate cell-to-cell communication, or quorum sensing (QS), in many bacterial species. A *P. aeruginosa hfq* mutant shows extensive alterations in the QS network, much of which occurs through Hfq’s effects on the RsmY sRNA (Sonnleitner et al., 2006). Hfq also contributes to PrrF’s ability to interact with the *antR* mRNA, which promotes the biosynthesis of multiple AQ metabolites including the Pseudomonas quinolone signal (PQS) (Djapgne et al., 2018), a QS molecule that activates the expression of multiple virulence genes (Collier et al., 2002). More recent work demonstrated that the PrrF sRNAs activate the expression of genes for T6SS *via* the positive impact on AQ synthesis (Brewer et al., 2020), suggesting Hfq may mediate additional effects on *P. aeruginosa* QS and virulence *via* the PrrF sRNAs.

Hfq-dependent sRNAs also play a key role in QS in *Vibrio* species. An initial screen for repressors of the QS system in *Vibrio harveyi* and *Vibrio cholerae* revealed the Hfq-dependent Qrr (quorum regulatory RNA) sRNAs, which were present in 4 to 5 copies and present in multiple pathogenic and nonpathogenic *Vibrio* species (Lenz et al., 2004). Regulation of at least one Qrr target mRNA (*hapR*) has been experimentally shown to occur through Hfq-dependent base-pairing (Bardill et al., 2011). The Qrr sRNAs also post-transcriptionally activate the expression of a diguanylate cyclase that is required for biofilm formation (Zhao et al., 2013), suggesting that the Hfq-Qrr regulatory network plays a central role in the sessile-motile lifestyle switch of *Vibrio* species.

### Pleiotropic Impacts of Hfq on Pathogenesis

As might be expected from the preceding sections, Hfq has a demonstrated requirement in virulence for many bacterial pathogens. Deletion of *hfq* in multiple *E. coli* pathovars, including uropathogenic *E. coli* (UPEC), enterohemorrhagic *E. coli* (EHEC), enteropathogenic *E. coli* (EPEC), causes reduced virulence (Simonsen et al., 2011; Bojer et al., 2012), and *hfq* mutants in these organisms show defects in multiple Hfq-dependent sRNA regulatory processes. In UPEC, this includes the loss of regulation by the PapR sRNA, which regulates P fimbriae needed for adherence (Khandige et al., 2015), induction of σ^E^ regulation, and loss of RpoS regulation (Kulesus et al., 2008). Since Hfq is required for RyhB stability in *E. coli* K12 (Masse et al., 2003), loss of *hfq* in UPEC is similarly expected to promote RyhB stability, thus further impacting siderophore production and virulence in UPEC. Notably, while *hfq* mutants in *E. coli* K12 and an EPEC strain both induced the σ^E^ pathway, the EPEC *hfq* mutant (and not *E. coli* K12) also induced the Cpx stress response due to dysregulation of an EPEC specific sRNA pathway responsible for pilus production (Vogt and Raivio, 2014). Moreover, Hfq in EHEC modulates expression of multiple additional virulence factors, including the locus of enterocyte effacement (LEE) locus, the Qse two component signaling system, and Shiga toxins (Hansen and Kaper, 2009; Shakhnovich et al., 2009; Kendall et al., 2011).

*V. cholerae hfq* mutants are also defective for virulence (Ding et al., 2004). Neither OMP nor envelope stress responses in *V. cholerae* were determined to be the source of virulence attenuation of the *V. cholerae hfq* mutant (Ding et al., 2004), and *ryhB* mutants showed no defect in virulence (Davis et al., 2005; Mey et al., 2005). It is therefore likely that the combined defects in multiple processes – including membrane integrity, biofilm formation, QS, and iron homeostasis – collectively contribute to the impact of Hfq on *V. cholerae* pathogenesis. A *P. aeruginosa hfq* mutant likewise exhibits pleiotropic effects on cell physiology, including virulence attenuation, growth defects, changes in quorum sensing, and upregulation of several PrrF-responsive genes including *antR* (Sonnleitner et al., 2003; Sonnleitner et al., 2006). This overlap suggests that attenuation of the *hfq* mutant is due at least in part to loss of the PrrF sRNAs; however, the impact of Hfq on *P. aeruginosa* physiology appears to be even more extensive than that of PrrF. Deletion of *hfq* similarly attenuated virulence in both *Salmonella* and *Yersinia*, presumably through multiple effects cell physiology (Sittka et al., 2007; Geng et al., 2009; Schiano et al., 2010; Deng et al., 2012; Lathem et al., 2014). Hfq is further required for virulence in additional Gram-negative pathogens, including *Burkholderia cepacia* (Sousa et al., 2010), *Bordetella pertussis* (Bibova et al., 2013), and *Borrelia burgdorferi* (Lybecker et al., 2010), and at least one Gram-positive pathogen, *Listeria monocytogenes* (Christiansen et al., 2004); however the specific roles that Hfq-dependent sRNA regulation play in these latter virulence phenotypes remain less clear.

While ascribing virulence defects to any one function of Hfq is complicated by its requirement for multiple sRNAs in the above species, additional factors can further confound understanding the attenuation of *hfq* mutants. First and foremost, many *hfq* mutants exhibit growth defects, which will affect survival in the host independently of any one specific sRNA function. Also important is the broad range of functions Hfq plays outside of sRNA regulation in many bacteria, including more newly understood roles in ribosomal biogenesis and protein translation [reviewed by (Dos Santos et al., 2019)]. Despite these caveats, Hfq-dependent sRNA regulation clearly plays a central and critical role in survival within the host for many bacterial pathogens.

## Additional sRNA Chaperone Proteins

While many bacterial sRNAs require Hfq for their function and stability, several notable exceptions exist. For instance, Hfq is present in *Staphylococcus aureus*, but does not appear to have any impact on sRNA function or regulation (Bohn et al., 2007). Moreover, the activity of orthologous sRNAs in related species often show varying dependencies on Hfq. This variation underlines the ambiguous requirement of Hfq for sRNA function and stability across prokaryotes. In light of this, several studies have identified additional chaperones that affect sRNA function in numerous bacterial species, though many species that express sRNAs still lack evidence for a *bona fide* RNA chaperone.

### FinO/ProQ Domain Chaperone Proteins

The chaperone protein ProQ was first identified in 1999 as an element in *E. coli* that influenced the osmotic activation of ProP at a posttranscriptional level (Kunte et al., 1999). ProP is an osmoregulatory transporter and is essential for solute movement across the plasma membrane (Kunte et al., 1999). Initial mutagenesis analyses revealed that mutation of the *proQ* sequence decreased the activation rate of the ProP protein but did not alter *proP* transcript or ProP protein levels (Kunte et al., 1999). Later studies suggested that wild type ProQ might control *proP* mRNA levels by acting as a chaperone protein (Chaulk et al., 2011). Additional investigations showed that ProQ interacts with two distinct *proP* mRNAs transcribed from different promoters, with equal affinities, and also interacts with the *rpoS* mRNA (Sheidy and Zielke, 2013). More recent analyses revealed that ProQ in *Salmonella* is a prominent binding partner of numerous sRNAs (Smirnov et al., 2016).

ProQ is a part of the FinO domain protein family, and recent structure studies show that ProQ’s N-terminal domain resembles that of the chaperone protein FinO, which regulates conjugation by modulating interactions between the *finP* sRNA and *traJ* mRNA (Arthur et al., 2011; Gonzalez et al., 2017). *In vitro* experiments showed that FinO recognizes single stranded regions on each of the RNA molecules, stabilizing the *finP* sRNA against degradation, and promoting the sRNA-mRNA interaction. Once stabilized, the *finP* and *traJ* RNAs base pair at a region of complementarity within their respective stem-loops, allowing RNase E to target the sRNA-mRNA complex for degradation and thus translation inhibition (Arthur et al., 2011). The ProQ/FinO domains have been structurally characterized across multiple Gram-negative species (*E. coli, Salmonella, Legionella pneumophilia*) and are generally shaped like a fist, with a conserved, positively charged RNA binding site on the concave side of the protein. This positively-charged region in one of two *L. pneumophilia* ProQ homologs, designated Lpp1663, showed a preference for binding single stranded U-rich RNA molecules (Immer et al., 2020). A second ProQ homolog in *L. pneumophilia*, originally designated Lpp0148 and re-named RocC, binds and stabilizes the RocR sRNA, which in turn represses mRNAs required for natural competence (Attaiech et al., 2016). RocC binds to RocR adjacent to a U-rich tail (Attaiech et al., 2016), seemingly in agreement with the sequence determinants identified for Lpp1663 RNA binding. RocR is the only binding partner for RocC that has yet been described, so it remains unclear how extensive ProQ homologs function as RNA chaperones in this and other pathogens where it has been identified.

In contrast, the ProQ proteins in *E. co*li and *Salmonella* clearly have extensive effects on sRNA regulatory networks, with some overlap with Hfq sRNA regulatory networks also being recently reported (Melamed et al., 2020). Moreover, *Salmonella* ProQ plays a central role in regulating factors required for pathogenicity. Specifically, a *∆proQ* mutant strain was defective in infecting HeLa cells as compared to the wild type strain, and the expression of virulence genes for motility, chemotaxis pathways, and numerous sigma factors was reduced in the ∆*proQ* mutant (Westermann et al., 2019). The levels of three ProQ-interacting sRNAs – SraL, RaiZ and STnc540 – were shown to be reduced in a ∆*proQ* mutant compared to the wild type strain when grown in HeLa cells (Westermann et al., 2019). Detailed analysis of the RaiZ sRNA showed that ProQ stabilizes RaiZ and promotes complex formation with the *hapR* mRNA, encoding histone-like protein (Smirnov et al., 2017). Moreover, levels of the STnc540 sRNA inversely correlated with that of the *mgtCBR* mRNA (Westermann et al., 2019), encoding a magnesium transport system that is required for survival inside macrophages (Blanc-Potard and Groisman, 1997). Deletion of *proQ* similarly affected levels of *mgtCBR* mRNA, indicating this mRNA is likely a direct target of ProQ-dependent STnc540 regulation (Blanc-Potard and Groisman, 1997). Two recent reports further demonstrated that FinO-dependent sRNAs regulate copy number of plasmids carrying antibiotic resistance genes in *E. coli* and *S. enterica* (El Mouali et al., 2021; Yang et al., 2021).

### The Fbp Proteins in *Bacillus subtilis*

As discussed above, the iron-regulated small basic proteins FbpA, FbpB, and FbpC work in collaboration with FsrA sRNA to mediate post-transcriptional gene regulation in *B. subtilis* (Smaldone et al., 2012). FsrA is expressed under low-iron environment and represses the synthesis of numerous iron-containing proteins such as the *lutABC* operon that encodes iron sulfur-containing proteins involved in lactate utilization (Smaldone et al., 2012). Several studies have established that these small proteins are required for FsrA sRNA stability and function by stabilizing and mediating the function of FsrA sRNA (Smaldone et al., 2012; Smaldone et al., 2012; Kim and Kwon, 2013). Based on these findings, it was hypothesized that FbpABC small proteins might behave as chaperone proteins, in a manner similar to how Hfq modulates regulation by iron-responsive sRNAs in Gram-negative bacteria.

## Concluding Statements

Even though many questions remain unanswered regarding the role of bacterial RNA chaperone proteins in gene expression and pathogenesis, advances in the field and the development of new methodologies over the past two decades have shed significant light on the matter. The more recently characterized ProQ as an sRNA chaperone is just one great example of our limited knowledge regarding the function of bacterial sRNAs. Identification and in depth characterization of sRNA chaperones and their mechanisms of action will open new avenues and provide better understanding of the regulation of bacterial virulence factors. Specific questions remaining to be answered include:

i) What are the specific mechanisms of Hfq-sRNA control of virulence in non-enteric bacteria?
ii) How do FinO/ProQ domain proteins contribute to virulence in non-enteric, as well as enteric, bacteria?
iii) Do other chaperones mediate sRNA regulation in organisms where Hfq or ProQ homologs are either lacking or play no discernable role in sRNA regulation?
iv) Or is sRNA regulation in some species capable of functioning in the absence of chaperone proteins?

Another intriguing area for study is determining how these sRNAs pathways have evolved from non-pathogenic species to related pathogens. For example, the increased acidity in the Hfq rim sequence in the more recently evolved *P. aeruginosa* (R16, K17, R19) from the non-pathogenic pseudomonads (K16, K17, K19) may shed light on sRNA regulatory mechanisms that are required for survival in the host. In this vein, continued characterization of already discovered RNA chaperone proteins and sRNA regulatory pathways amongst highly related species may reveal new appreciation for sRNA mechanisms that are required for virulence.

## Author Contributions

LD wrote the majority of the review as a part of her thesis work, with edits contributed by AO. All authors contributed to the article and approved the submitted version.

## Funding

This work was supported by NIH Grant 1R01 AI123320 (AO).

## Conflict of Interest

The authors declare that the research was conducted in the absence of any commercial or financial relationships that could be construed as a potential conflict of interest.

## References

[B1] Aiba H. (2007). Mechanism of RNA Silencing by Hfq-Binding Small RNAs. Curr. Opin. Microbiol. 10, 134–139. 10.1016/j.mib.2007.03.010 17383928

[B2] Argaman L. Elgrably-Weiss M. Hershko T. Vogel J. Altuvia S. (2012). Rela Protein Stimulates the Activity of RyhB Small RNA by Acting on RNA-Binding Protein Hfq. Proc. Natl. Acad. Sci. U. S. A. 109, 4621–4626. 10.1073/pnas.1113113109 22393021 PMC3311362

[B3] Arluison V. Hohng S. Roy R. Pellegrini O. Regnier P. Ha T. (2007). Spectroscopic Observation of RNA Chaperone Activities of Hfq in Post-Transcriptional Regulation by a Small Non-Coding RNA. Nucleic Acids Res. 35, 999–1006. 10.1093/nar/gkl1124 17259214 PMC1807976

[B4] Arthur D. C. Edwards R. A. Tsutakawa S. Tainer J. A. Frost L. S. Glover J. N. (2011). Mapping Interactions Between the RNA Chaperone FinO and Its RNA Targets. Nucleic Acids Res. 39, 4450–4463. 10.1093/nar/gkr025 21278162 PMC3105414

[B5] Attaiech L. Boughammoura A. Brochier-Armanet C. Allatif O. Peillard-Fiorente F. Edwards R. A.. (2016). Silencing of Natural Transformation by an RNA Chaperone and a Multitarget Small RNA. Proc. Natl. Acad. Sci. U. S. A. 113, 8813–8818. 10.1073/pnas.1601626113 27432973 PMC4978251

[B6] Babitzke P. Romeo T. (2007). Csrb sRNA Family: Sequestration of RNA-Binding Regulatory Proteins. Curr. Opin. Microbiol. 10, 156–163. 10.1016/j.mib.2007.03.007 17383221

[B7] Bardill J. P. Zhao X. Hammer B. K. (2011). The *Vibrio cholerae* Quorum Sensing Response Is Mediated by Hfq-Dependent sRNA/mRNA Base Pairing Interactions. Mol. Microbiol. 80, 1381–1394. 10.1111/j.1365-2958.2011.07655.x 21453446

[B8] Beisel C. L. Updegrove T. B. Janson B. J. Storz G. (2012). Multiple Factors Dictate Target Selection by Hfq-Binding Small RNAs. EMBO J. 31, 1961–1974. 10.1038/emboj.2012.52 22388518 PMC3343335

[B9] Bibova I. Skopova K. Masin J. Cerny O. Hot D. Sebo P.. (2013). The RNA Chaperone Hfq Is Required for Virulence of *Bordetella pertussis* . Infect. Immun. 81, 4081–4090. 10.1128/IAI.00345-13 23980112 PMC3811842

[B10] Blanc-Potard A. B. Groisman E. A. (1997). The Salmonella SelC Locus Contains a Pathogenicity Island Mediating Intramacrophage Survival. EMBO J. 16, 5376–5385. 10.1093/emboj/16.17.5376 9311997 PMC1170169

[B11] Bohn C. Rigoulay C. Bouloc P. (2007). No Detectable Effect of RNA-Binding Protein Hfq Absence in *Staphylococcus aureus* . BMC Microbiol. 7, 10. 10.1186/1471-2180-7-10 17291347 PMC1800855

[B12] Bojer M. S. Jakobsen H. Struve C. Krogfelt K. A. Lobner-Olesen A. (2012). Lack of the RNA Chaperone Hfq Attenuates Pathogenicity of Several *Escherichia coli* Pathotypes Towards Caenorhabditis Elegans. Microbes Infect. 14, 1034–1039. 10.1016/j.micinf.2012.06.002 22713744

[B13] Bossi L. Figueroa-Bossi N. (2007). A Small RNA Downregulates LamB Maltoporin in Salmonella. Mol. Microbiol. 65, 799–810. 10.1111/j.1365-2958.2007.05829.x 17608792

[B14] Brady R. A. Leid J. G. Calhoun J. H. Costerton J. W. Shirtliff M. E. (2008). Osteomyelitis and the Role of Biofilms in Chronic Infection. FEMS Immunol. Med. Microbiol. 52, 13–22. 10.1111/j.1574-695X.2007.00357.x 18081847

[B15] Brewer L. K. Huang W. Hackert B. J. Kane M. A. Oglesby A. G. (2020). Static Growth Promotes PrrF and 2-Alkyl-4(1H)-Quinolone Regulation of Type Vi Secretion Protein Expression in *Pseudomonas aeruginosa* . J. Bacteriol. 202, e00416-20. 10.1128/JB.00416-20 PMC7685562 33020221

[B16] Broach W. H. Egan N. Wing H. J. Payne S. M. Murphy E. R. (2012). Virf-Independent Regulation of Shigella Virb Transcription Is Mediated by the Small RNA RyhB. PloS One 7, e38592. 10.1371/journal.pone.0038592 22701677 PMC3372517

[B17] Brown L. Elliott T. (1996). Efficient Translation of the Rpos Sigma Factor in Salmonella Typhimurium Requires Host Factor I, An RNA-Binding Protein Encoded by the Hfq Gene. J. Bacteriol. 178, 3763–3770. 10.1128/jb.178.13.3763-3770.1996 8682778 PMC232634

[B18] Chakravarty S. Masse E. (2019). RNA-Dependent Regulation of Virulence in Pathogenic Bacteria. Front. Cell. Infection Microbiol. 9, 337. 10.3389/fcimb.2019.00337 PMC6794450 31649894

[B19] Chatterjee A. Cui Y. Chatterjee A. K. (2002). Rsma and the Quorum-Sensing Signal, N-3-Oxohexanoyl.-L-Homoserine Lactone, Control the Levels of Rsmb RNA in Erwinia Carotovora Subsp. Carotovora by Affecting Its Stability. J. Bacteriol. 184, 4089–4095. 10.1128/JB.184.15.4089-4095.2002 12107125 PMC135201

[B20] Chatterjee Y. C. A. Liu Y. Dumenyo C. K. Chatterjee A. K. (1995). Inactivation of Rsma Leads to Overproduction of Extracellular Pectinases, Cellulases, and Proteases in Erwinia Carotovora Subsp. Carotovora in the Absence of the Starvation/Cell Density-Sensing Signal, N-(3-Oxohexanoyl)-L-Homoserine Lactone. ASM Appl Environ Microbiol. 61 (5), 1959–67. 10.1128/aem.61.5.1959-1967.1995 PMC167458 7646031

[B21] Chaulk S. G. Smith Frieday M. N. Arthur D. C. Culham D. E. Edwards R. A. Soo P.. (2011). ProQ Is an RNA Chaperone That Controls ProP Levels in *Escherichia coli* . Biochemistry 50, 3095–3106. 10.1021/bi101683a 21381725

[B22] Christiansen J. K. Larsen M. H. Ingmer H. Sogaard-Andersen L. Kallipolitis B. H. (2004). The RNA-Binding Protein Hfq of Listeria Monocytogenes: Role in Stress Tolerance and Virulence. J. Bacteriol. 186, 3355–3362. 10.1128/JB.186.11.3355-3362.2004 15150220 PMC415768

[B23] Collier D. N. Anderson L. McKnight S. L. Noah T. L. Knowles M. Boucher R.. (2002). A Bacterial Cell to Cell Signal in the Lungs of Cystic Fibrosis Patients. FEMS Microbiol. Lett. 215, 41–46. 10.1111/j.1574-6968.2002.tb11367.x 12393198

[B24] Costerton J. W. Stewart P. S. (2001). Battling Biofilms. Sci. Am. 285, 74–81. 10.1038/scientificamerican0701-74 11432197

[B25] Davis B. M. Quinones M. Pratt J. Ding Y. Waldor M. K. (2005). Characterization of the Small Untranslated RNA RyhB and Its Regulon in *Vibrio Cholerae* . J. Bacteriol. 187, 4005–4014. 10.1128/JB.187.12.4005-4014.2005 15937163 PMC1151736

[B26] Deng Z. Meng X. Su S. Liu Z. Ji X. Zhang Y.. (2012). Two sRNA RyhB Homologs From *Yersinia pestis* Biovar Microtus Expressed *In Vivo* Have Differential Hfq-Dependent Stability. Res. Microbiol. 163, 413–418. 10.1016/j.resmic.2012.05.006 22659336

[B27] Ding Y. Davis B. M. Waldor M. K. (2004). Hfq Is Essential for *Vibrio cholerae* Virulence and Downregulates Sigma Expression. Mol. Microbiol. 53, 345–354. 10.1111/j.1365-2958.2004.04142.x 15225327

[B28] Djapgne L. Panja S. Brewer L. Gans J. Kane M. A. Woodson S. A.. (2018). The *Pseudomonas aeruginosa* PrrF1 and PrrF2 Small Regulatory RNAs (sRNAs) Promote 2-Alkyl-4-Quinolone Production Through Redundant Regulation of the Antr mRNA. J. Bacteriol. 200 (10), e00704-17. 10.1128/JB.00704-17 29507088 PMC5915787

[B29] Dos Santos R. F. Arraiano C. M. Andrade J. M. (2019). New Molecular Interactions Broaden the Functions of the RNA Chaperone Hfq. Curr. Genet. 65, 1313–1319. 10.1007/s00294-019-00990-y 31104083

[B30] Ducey T. F. Jackson L. Orvis J. Dyer D. W. (2009). Transcript Analysis of *NrrF*, A Fur Repressed sRNA of Neisseria Gonorrhoeae. Microbial Pathogenesis 46, 166–170. 10.1016/j.micpath.2008.12.003 19162160 PMC4890603

[B31] Elisabeth Sonnleitner L. A. Haas D. (2009). Small RNA as Global Regulator of Carbon Catabolite Repression in Pseudomonas Aeruginosa. Proc. Natl. Acad. Sci. U. S. A. 06 (51), 21866–21871. 10.1073/pnas.0910308106 PMC2799872 20080802

[B32] El Mouali Y. Gerovac M. Mineikaite R. Vogel J. (2021). In Vivo Targets of Salmonella FinO Include a Finp-Like Small RNA Controlling Copy Number of a Cohabitating Plasmid. Nucleic Acids Res. 49, 5319–5335. 10.1093/nar/gkab281 33939833 PMC8136791

[B33] Fender A. Elf J. Hampel K. Zimmermann B. Wagner E. G. (2010). RNAs Actively Cycle on the Sm-Like Protein Hfq. Genes Dev. 24, 2621–2626. 10.1101/gad.591310 21123649 PMC2994036

[B34] Figueroa-Bossi N. Lemire S. Maloriol D. Balbontin R. Casadesus J. Bossi L. (2006). Loss of Hfq Activates the SigmaE-Dependent Envelope Stress Response in Salmonella Enterica. Mol. Microbiol. 62, 838–852. 10.1111/j.1365-2958.2006.05413.x 16999834

[B35] Franze de Fernandez M. T. Eoyang L. August J. T. (1968). Factor Fraction Required for the Synthesis of Bacteriophage Qbeta-RNA. Nature 219, 588–590. 10.1038/219588a0 4874917

[B36] Geng J. Song Y. Yang L. Feng Y. Qiu Y. Li G.. (2009). Involvement of the Post-Transcriptional Regulator Hfq in *Yersinia pestis* Virulence. PloS One 4, e6213. 10.1371/journal.pone.0006213 19593436 PMC2704395

[B37] Gonzalez G. M. Hardwick S. W. Maslen S. L. Skehel J. M. Holmqvist E. Vogel J.. (2017). Structure of the *Escherichia coli* ProQ RNA-Binding Protein. RNA 23, 696–711. 10.1261/rna.060343.116 28193673 PMC5393179

[B38] Guisbert E. Rhodius V. A. Ahuja N. Witkin E. Gross C. A. (2007). Hfq Modulates the SigmaE-Mediated Envelope Stress Response and the Sigma32-Mediated Cytoplasmic Stress Response in *Escherichia coli* . J. Bacteriol. 189, 1963–1973. 10.1128/JB.01243-06 17158661 PMC1855744

[B39] Guo M. S. Updegrove T. B. Gogol E. B. Shabalina S. A. Gross C. A. Storz G. (2014). Micl, a New SigmaE-Dependent sRNA, Combats Envelope Stress by Repressing Synthesis of Lpp, the Major Outer Membrane Lipoprotein. Genes Dev. 28, 1620–1634. 10.1101/gad.243485.114 25030700 PMC4102768

[B40] Hansen A. M. Kaper J. B. (2009). Hfq Affects the Expression of the LEE Pathogenicity Island in Enterohaemorrhagic *Escherichia coli* . Mol. Microbiol. 73, 446–465. 10.1111/j.1365-2958.2009.06781.x 19570135 PMC2770234

[B41] Hoekzema M. Romilly C. Holmqvist E. Wagner E. G. H. (2019). Hfq-Dependent mRNA Unfolding Promotes sRNA-Based Inhibition of Translation. EMBO J. 38, e101199. 10.15252/embj.2018101199 30833291 PMC6443205

[B42] Hoiby N. Ciofu O. Johansen H. K. Song Z. J. Moser C. Jensen P. O.. (2011). The Clinical Impact of Bacterial Biofilms. Int. J. Oral. Sci. 3, 55–65. 10.4248/IJOS11026 21485309 PMC3469878

[B43] Hopkins J. F. Panja S. Woodson S. A. (2011). Rapid Binding and Release of Hfq From Ternary Complexes During RNA Annealing. Nucleic Acids Res. 39, 5193–5202. 10.1093/nar/gkr062 21378124 PMC3130257

[B44] Horstmann N. Orans J. Valentin-Hansen P. Shelburne S. A. 3rd Brennan R. G. (2012). Structural Mechanism of Staphylococcus Aureus Hfq Binding to an RNA a-Tract. Nucleic Acids Res. 40, 11023–11035. 10.1093/nar/gks809 22965117 PMC3505971

[B45] Hwang W. Arluison V. Hohng S. (2011). Dynamic Competition of Dsra and Rpos Fragments for the Proximal Binding Site of Hfq as a Means for Efficient Annealing. Nucleic Acids Res. 39, 5131–5139. 10.1093/nar/gkr075 21357187 PMC3130260

[B46] Ikeda Y. Yagi M. Morita T. Aiba H. (2011). Hfq Binding at Rhlb-Recognition Region of RNase E Is Crucial for the Rapid Degradation of Target mRNAs Mediated by sRNAs in *Escherichia coli* . Mol. Microbiol. 79, 419–432. 10.1111/j.1365-2958.2010.07454.x 21219461

[B47] Immer C. Hacker C. Wohnert J. (2020). Solution Structure and RNA-Binding of a Minimal ProQ-Homolog From *Legionella pneumophila* (Lpp1663). RNA 26, 2031–2043. 10.1261/rna.077354.120 32989045 PMC7668265

[B48] Irie Y. La Mensa A. Murina V. Hauryliuk V. Tenson T. Shingler V. (2020). Hfq-Assisted Rsma Regulation Is Central to *Pseudomonas aeruginosa* Biofilm Polysaccharide PEL Expression. Front. Microbiol. 11, 482585. 10.3389/fmicb.2020.482585 33281751 PMC7705225

[B49] Jackson L. A. Pan J. C. Day M. W. Dyer D. W. (2013). Control of RNA Stability by Nrrf, an Iron-Regulated Small RNA in Neisseria Gonorrhoeae. J. Bacteriol. 10.1128/JB.00839-13 PMC3811587 24039262

[B50] Janssen K. H. Diaz M. R. Gode C. J. Wolfgang M. C. Yahr T. L. (2018). Rsmv, a Small Noncoding Regulatory RNA in *Pseudomonas aeruginosa* That Sequesters Rsma and Rsmf From Target mRNAs. J. Bacteriol. 200, 00277-18. 10.1128/JB.00277-18 PMC6060366 29866805

[B51] Johansen J. Eriksen M. Kallipolitis B. Valentin-Hansen P. (2008). Down-Regulation of Outer Membrane Proteins by Noncoding RNAs: Unraveling the Camp-CRP- and SigmaE-Dependent CyaR-Ompx Regulatory Case. J. Mol. Biol. 383, 1–9. 10.1016/j.jmb.2008.06.058 18619465

[B52] Johansen J. Rasmussen A. A. Overgaard M. Valentin-Hansen P. (2006). Conserved Small Non-Coding RNAs That Belong to the SigmaE Regulon: Role in Down-Regulation of Outer Membrane Proteins. J. Mol. Biol. 364, 1–8. 10.1016/j.jmb.2006.09.004 17007876

[B53] Kendall M. M. Gruber C. C. Rasko D. A. Hughes D. T. Sperandio V. (2011). Hfq Virulence Regulation in Enterohemorrhagic *Escherichia coli* O157:H7 Strain 86-24. J. Bacteriol. 193, 6843–6851. 10.1128/JB.06141-11 21984790 PMC3232842

[B54] Khandige S. Kronborg T. Uhlin B. E. Møller-Jensen J. (2015). sRNA-Mediated Regulation of P-Fimbriae Phase Variation in Uropathogenic *Escherichia coli* . PloS Pathog. 11, e1005109. 10.1371/journal.ppat.1005109 26291711 PMC4546395

[B55] Kim J. N. Kwon Y. M. (2013). Genetic and Phenotypic Characterization of the RyhB Regulon in Salmonella Typhimurium. Microbiol. Res. 168, 41–49. 10.1016/j.micres.2012.06.007 22824499

[B56] Kint G. De Coster D. Marchal K. Vanderleyden J. De Keersmaecker S. C. (2010). The Small Regulatory RNA Molecule Mica Is Involved in Salmonella Enterica Serovar Typhimurium Biofilm Formation. BMC Microbiol. 10, 276. 10.1186/1471-2180-10-276 21044338 PMC2987988

[B57] Klein G. Raina S. (2017). Small Regulatory Bacterial RNAs Regulating the Envelope Stress Response. Biochem. Soc. Trans. 45, 417–425. 10.1042/BST20160367 28408482 PMC5736990

[B58] Kulesus R. R. Diaz-Perez K. Slechta E. S. Eto D. S. Mulvey M. A. (2008). Impact of the RNA Chaperone Hfq on the Fitness and Virulence Potential of Uropathogenic *Escherichia coli* . Infect. Immun. 76, 3019–3026. 10.1128/IAI.00022-08 18458066 PMC2446724

[B59] Kunte H. J. Crane R. A. Culham D. E. Richmond D. Wood J. M. (1999). Protein ProQ Influences Osmotic Activation of Compatible Solute Transporter ProP in *Escherichia coli* K-12. J. Bacteriol. 181, 1537–1543. 10.1128/JB.181.5.1537-1543.1999 10049386 PMC93544

[B60] Kwiatkowska J. Wroblewska Z. Johnson K. A. Olejniczak M. (2018). The Binding of Class II sRNA Mgrr to Two Different Sites on Matchmaker Protein Hfq Enables Efficient Competition for Hfq and Annealing to Regulated mRNAs. RNA 24, 1761–1784. 10.1261/rna.067777.118 30217864 PMC6239178

[B61] Lapouge K. Schubert M. Allain F. H. Haas D. (2008). Gac/Rsm Signal Transduction Pathway of Gamma-Proteobacteria: From RNA Recognition to Regulation of Social Behaviour. Mol. Microbiol. 67, 241–253. 10.1111/j.1365-2958.2007.06042.x 18047567

[B62] Lathem W. W. Schroeder J. A. Bellows L. E. Ritzert J. T. Koo J. T. Price P. A.. (2014). Posttranscriptional Regulation of the *Yersinia pestis* Cyclic AMP Receptor Protein Crp and Impact on Virulence. mBio 5, e01038–e01013. 10.1128/mBio.01038-13 24520064 PMC3950509

[B63] Leclerc J. M. Dozois C. M. Daigle F. (2013). Role of the Salmonella Enterica Serovar Typhi Fur Regulator and Small RNAs Rfra and Rfrb in Iron Homeostasis and Interaction With Host Cells. Microbiol. (Reading) 159, 591–602. 10.1099/mic.0.064329-0 23306672

[B64] Leng Y. Vakulskas C. A. Zere T. R. Pickering B. S. Watnick P. I. Babitzke P.. (2016). Regulation of Csrb/C sRNA Decay by EIIA(Glc) of the Phosphoenolpyruvate: Carbohydrate Phosphotransferase System. Mol. Microbiol. 99, 627–639. 10.1111/mmi.13259 26507976 PMC4888959

[B65] Lenz D. H. Miller M. B. Zhu J. Kulkarni R. V. Bassler B. L. (2005). CsrA and Three Redundant Small RNAs Regulate Quorum Sensing in *Vibrio cholerae* . Mol. Microbiol. 58, 1186–1202. 10.1111/j.1365-2958.2005.04902.x 16262799

[B66] Lenz D. H. Mok K. C. Lilley B. N. Kulkarni R. V. Wingreen N. S. Bassler B. L. (2004). The Small RNA Chaperone Hfq and Multiple Small RNAs Control Quorum Sensing in Vibrio Harveyi and *Vibrio cholerae* . Cell 118, 69–82. 10.1016/j.cell.2004.06.009 15242645

[B67] Lybecker M. C. Abel C. A. Feig A. L. Samuels D. S. (2010). Identification and Function of the RNA Chaperone Hfq in the Lyme Disease Spirochete Borrelia Burgdorferi. Mol. Microbiol. 78, 622–635. 10.1111/j.1365-2958.2010.07374.x 20815822 PMC2963666

[B68] Masse E. Escorcia F. E. Gottesman S. (2003). Coupled Degradation of a Small Regulatory RNA and Its mRNA Targets in *Escherichia coli* . Genes Dev. 17, 2374–2383. 10.1101/gad.1127103 12975324 PMC218075

[B69] Masse E. Gottesman S. (2002). A Small RNA Regulates the Expression of Genes Involved in Iron Metabolism in *Escherichia coli* . Proc. Natl. Acad. Sci. U. S. A. 99, 4620–4625. 10.1073/pnas.032066599 11917098 PMC123697

[B70] Melamed S. Adams P. P. Zhang A. Zhang H. Storz G. (2020). RNA-RNA Interactomes of ProQ and Hfq Reveal Overlapping and Competing Roles. Mol. Cell 77, 411–425.e7. 10.1016/j.molcel.2019.10.022 31761494 PMC6980735

[B71] Mellin J. R. Goswami S. Grogan S. Tjaden B. Genco C. A. (2007). A Novel Fur- and Iron-Regulated Small RNA, Nrrf, Is Required for Indirect Fur-Mediated Regulation of the Sdha and Sdhc Genes in Neisseria Meningitidis. J. Bacteriol. 189, 3686–3694. 10.1128/JB.01890-06 17351036 PMC1913314

[B72] Metruccio M. M. Fantappie L. Serruto D. Muzzi A. Roncarati D. Donati C.. (2009). The Hfq-Dependent Small Noncoding RNA Nrrf Directly Mediates Fur-Dependent Positive Regulation of Succinate Dehydrogenase in Neisseria Meningitidis. J. Bacteriol. 191, 1330–1342. 10.1128/JB.00849-08 19060140 PMC2631994

[B73] Mey A. R. Craig S. A. Payne S. M. (2005). Characterization of *Vibrio cholerae* RyhB: The RyhB Regulon and Role of RyhB in Biofilm Formation. Infect. Immun. 73, 5706–5719. 10.1128/IAI.73.9.5706-5719.2005 16113288 PMC1231101

[B74] Milojevic T. Grishkovskaya I. Sonnleitner E. Djinovic-Carugo K. Blasi U. (2013). The *Pseudomonas aeruginosa* Catabolite Repression Control Protein Crc Is Devoid of RNA Binding Activity. PloS One 8, e64609. 10.1371/journal.pone.0064609 23717639 PMC3662782

[B75] Mohanty B. K. Maples V. F. Kushner S. R. (2004). The Sm-Like Protein Hfq Regulates Polyadenylation Dependent mRNA Decay in *Escherichia coli* . Mol. Microbiol. 54, 905–920. 10.1111/j.1365-2958.2004.04337.x 15522076

[B76] Monteiro C. Papenfort K. Hentrich K. Ahmad I. Le Guyon S. Reimann R.. (2012). Hfq and Hfq-Dependent Small RNAs Are Major Contributors to Multicellular Development in Salmonella Enterica Serovar Typhimurium. RNA Biol. 9, 489–502. 10.4161/rna.19682 22336758

[B77] Moreno R. Hernandez-Arranz S. La Rosa R. Yuste L. Madhushani A. Shingler V.. (2015). The Crc and Hfq Proteins of Pseudomonas Putida Cooperate in Catabolite Repression and Formation of Ribonucleic Acid Complexes With Specific Target Motifs. Environ. Microbiol. 17, 105–118. 10.1111/1462-2920.12499 24803210

[B78] Moreno R. Ruiz-Manzano A. Yuste L. Rojo F. (2007). The Pseudomonas Putida Crc Global Regulator Is an RNA Binding Protein That Inhibits Translation of the Alks Transcriptional Regulator. Mol. Microbiol. 64, 665–675. 10.1111/j.1365-2958.2007.05685.x 17462015

[B79] Nelson C. E. Huang W. Brewer L. K. Nguyen A. T. Kane M. A. Wilks A.. (2019). Proteomic Analysis of the *Pseudomonas aeruginosa* Iron Starvation Response Reveals PrrF Small Regulatory RNA-Dependent Iron Regulation of Twitching Motility, Amino Acid Metabolism, and Zinc Homeostasis Proteins. J. Bacteriol. 201. 10.1128/JB.00754-18 PMC6531625 30962354

[B80] Nikulin A. Stolboushkina E. Perederina A. Vassilieva I. Blaesi U. Moll I.. (2005). Structure of *Pseudomonas aeruginosa* Hfq Protein. Acta Crystallogr. D Biol. Crystallogr. 61, 141–146. 10.1107/S0907444904030008 15681864

[B81] Nishijyo T. Haas D. Itoh Y. (2001). The Cbra-Cbrb Two-Component Regulatory System Controls the Utilization of Multiple Carbon and Nitrogen Sources in *Pseudomonas aeruginosa* . Mol. Microbiol. 40, 917–931. 10.1046/j.1365-2958.2001.02435.x 11401699

[B82] Oglesby A. G. Murphy E. R. Iyer V. R. Payne S. M. (2005). Fur Regulates Acid Resistance in Shigella Flexneri *Via* RyhB and Ydep. Mol. Microbiol. 58, 1354–1367. 10.1111/j.1365-2958.2005.04920.x 16313621

[B83] Oglesby-Sherrouse A. G. Murphy E. R. (2013). Iron-Responsive Bacterial Small RNas: Variations on a Theme. Metallomics: Integrated Biometal Sci. 5, 276–286. 10.1039/c3mt20224k PMC3612141 23340911

[B84] Olejniczak M. Storz G. (2017). ProQ/FinO-Domain Proteins: Another Ubiquitous Family of RNA Matchmakers? Mol. Microbiol. 104, 905–915. 10.1111/mmi.13679 28370625 PMC5578414

[B85] Panja S. Schu D. J. Woodson S. A. (2013). Conserved Arginines on the Rim of Hfq Catalyze Base Pair Formation and Exchange. Nucleic Acids Res. 41, 7536–7546. 10.1093/nar/gkt521 23771143 PMC3753642

[B86] Pannekoek Y. Huis In ‘t Veld R. Schipper K. Bovenkerk S. Kramer G. Speijer D.. (2017). Regulation of. FEBS Open Bio 7, 1302–1315. 10.1002/2211-5463.12266 PMC5586341 28904860

[B87] Papenfort K. Pfeiffer V. Lucchini S. Sonawane A. Hinton J. C. Vogel J. (2008). Systematic Deletion of Salmonella Small RNA Genes Identifies CyaR, a Conserved CRP-Dependent Riboregulator of Ompx Synthesis. Mol. Microbiol. 68, 890–906. 10.1111/j.1365-2958.2008.06189.x 18399940

[B88] Pei X. Y. Dendooven T. Sonnleitner E. Chen S. Blasi U. Luisi B. F. (2019). Architectural Principles for Hfq/Crc-Mediated Regulation of Gene Expression. Elife 8, e43158. 10.7554/eLife.43158 30758287 PMC6422490

[B89] Peng Y. Curtis J. E. Fang X. Woodson S. A. (2014). Structural Model of an mRNA in Complex With the Bacterial Chaperone Hfq. Proc. Natl. Acad. Sci. U .S. A. 111, 17134–17139. 10.1073/pnas.1410114111 25404287 PMC4260595

[B90] Peng Y. Soper T. J. Woodson S. A. (2014). Positional Effects of AAN Motifs in Rpos Regulation by sRNAs and Hfq. J. Mol. Biol. 426, 275–285. 10.1016/j.jmb.2013.08.026 24051417 PMC3947347

[B91] Porcheron G. Habib R. Houle S. Caza M. Lepine F. Daigle F.. (2014). The Small RNA RyhB Contributes to Siderophore Production and Virulence of Uropathogenic *Escherichia coli* . Infect. Immun. 82, 5056–5068. 10.1128/IAI.02287-14 25245805 PMC4249264

[B92] Prevost K. Salvail H. Desnoyers G. Jacques J. F. Phaneuf E. Masse E. (2007). The Small RNA RyhB Activates the Translation of Shia mRNA Encoding a Permease of Shikimate, a Compound Involved in Siderophore Synthesis. Mol. Microbiol. 64, 1260–1273. 10.1111/j.1365-2958.2007.05733.x 17542919

[B93] Quendera A. P. Seixas A. F. Dos Santos R. F. Santos I. Silva J. P. N. Arraiano C. M.. (2020). RNA-Binding Proteins Driving the Regulatory Activity of Small Non-Coding RNAs in Bacteria. Front. Mol. Biosci. 7, 78. 10.3389/fmolb.2020.00078 32478092 PMC7237705

[B94] Reimmann C. Valverde C. Kay E. Haas D. (2005). Posttranscriptional Repression of Gacs/Gaca-Controlled Genes by the RNA-Binding Protein Rsme Acting Together With Rsma in the Biocontrol Strain Pseudomonas Fluorescens CHA0. J. Bacteriol. 187, 276–285. 10.1128/JB.187.1.276-285.2005 15601712 PMC538806

[B95] Reinhart A. A. Nguyen A. T. Brewer L. K. Bevere J. Jones J. W. Kane M. A.. (2017). The *Pseudomonas aeruginosa* PrrF Small RNAs Regulate Iron Homeostasis During Acute Murine Lung Infection. Infect. Immun. 85, e00764-16. 10.1128/IAI.00764-16 28289146 PMC5400841

[B96] Reinhart A. A. Powell D. A. Nguyen A. T. O’Neill M. Djapgne L. Wilks A.. (2015). The PrrF-Encoded Small Regulatory RNAs Are Required for Iron Homeostasis and Virulence of *Pseudomonas aeruginosa* . Infect. Immun. 83, 863–875. 10.1128/IAI.02707-14 25510881 PMC4333466

[B97] Rojo F. (2010). Carbon Catabolite Repression in Pseudomonas: Optimizing Metabolic Versatility and Interactions With the Environment. FEMS Microbiol. Rev. 34, 658–684. 10.1111/j.1574-6976.2010.00218.x 20412307

[B98] Rollauer S. E. Sooreshjani M. A. Noinaj N. Buchanan S. K. (2015). Outer Membrane Protein Biogenesis in Gram-Negative Bacteria. Philos. Trans. R Soc. Lond B Biol. Sci. 370, 20150023. 10.1098/rstb.2015.0023 26370935 PMC4632599

[B99] Romero M. Miller C. L. Rajasekhar Karna S. L. Chen T. Heeb S. Leung K. P. (2016). RsmW, *Pseudomonas aeruginosa* Small Non-Coding Rsma-Binding RNA Upregulated in Biofilm Versus Planktonic Growth Conditions. BMC Microbiol. 10.1186/s12866-016-0771-y PMC4950607 27430253

[B100] Santana E. A. Harrison A. Zhang X. Baker B. D. Kelly B. J. White P.. (2014). Hrrf Is the Fur-Regulated Small RNA in Nontypeable Haemophilus Influenzae. PloS One 9, e105644. 10.1371/journal.pone.0105644 25157846 PMC4144887

[B101] Santiago-Frangos A. Frohlich K. S. Jeliazkov J. R. Malecka E. M. Marino G. Gray J. J.. (2019). Caulobacter Crescentus Hfq Structure Reveals a Conserved Mechanism of RNA Annealing Regulation. Proc. Natl. Acad. Sci. U. S. A. 116, 10978–10987. 10.1073/pnas.1814428116 31076551 PMC6561178

[B102] Sass A. M. Coenye T. (2020). Low Iron-Induced Small RNA Brrf Regulates Central Metabolism and Oxidative Stress Responses in Burkholderia Cenocepacia. PloS One 15, e0236405. 10.1371/journal.pone.0236405 32702060 PMC7377471

[B103] Schiano C. A. Bellows L. E. Lathem W. W. (2010). The Small RNA Chaperone Hfq Is Required for the Virulence of Yersinia Pseudotuberculosis. Infect. Immun. 78, 2034–2044. 10.1128/IAI.01046-09 20231416 PMC2863511

[B104] Schu D. J. Zhang A. Gottesman S. Storz G. (2015). Alternative Hfq-sRNA Interaction Modes Dictate Alternative mRNA Recognition. EMBO J. 34, 2557–2573. 10.15252/embj.201591569 26373314 PMC4609186

[B105] Shakhnovich E. A. Davis B. M. Waldor M. K. (2009). Hfq Negatively Regulates Type III Secretion in EHEC and Several Other Pathogens. Mol. Microbiol. 74, 347–363. 10.1111/j.1365-2958.2009.06856.x 19703108 PMC2765575

[B106] Sheidy D. T. Zielke R. A. (2013). Analysis and Expansion of the Role of the *Escherichia coli* Protein ProQ. PloS One 8, e79656. 10.1371/journal.pone.0079656 24205389 PMC3808355

[B107] Simonsen K. T. Nielsen G. Bjerrum J. V. Kruse T. Kallipolitis B. H. Moller-Jensen J. (2011). A Role for the RNA Chaperone Hfq in Controlling Adherent-Invasive *Escherichia coli* Colonization and Virulence. PloS One 6, e16387. 10.1371/journal.pone.0016387 21298102 PMC3027648

[B108] Sittka A. Pfeiffer V. Tedin K. Vogel J. (2007). The RNA Chaperone Hfq Is Essential for the Virulence of Salmonella Typhimurium. Mol. Microbiol. 63, 193–217. 10.1111/j.1365-2958.2006.05489.x 17163975 PMC1810395

[B109] Smaldone G. T. Antelmann H. Gaballa A. Helmann J. D. (2012a). The Fsra sRNA and Fbpb Protein Mediate the Iron-Dependent Induction of the *Bacillus subtilis* Lutabc Iron-Sulfur-Containing Oxidases. J. Bacteriol. 194, 2586–2593. 10.1128/JB.05567-11 22427629 PMC3347220

[B110] Smaldone G. T. Revelles O. Gaballa A. Sauer U. Antelmann H. Helmann J. D. (2012b). A Global Investigation of the Bacillus Subtilis Iron-Sparing Response Identifies Major Changes in Metabolism. J. Bacteriol. 194, 2594–2605. 10.1128/JB.05990-11 22389480 PMC3347201

[B111] Smirnov A. Forstner K. U. Holmqvist E. Otto A. Gunster R. Becher D.. (2016). Grad-Seq Guides the Discovery of ProQ as a Major Small RNA-Binding Protein. Proc. Natl. Acad. Sci. U. S. A. 113, 11591–11596. 10.1073/pnas.1609981113 27671629 PMC5068311

[B112] Smirnov A. Wang C. Drewry L. L. Vogel J. (2017). Molecular Mechanism of mRNA Repression in Trans by a ProQ-Dependent Small RNA. EMBO J. 36, 1029–1045. 10.15252/embj.201696127 28336682 PMC5391140

[B113] Someya T. Baba S. Fujimoto M. Kawai G. Kumasaka T. Nakamura K. (2012). Crystal Structure of Hfq From Bacillus Subtilis in Complex With SELEX-Derived RNA Aptamer: Insight Into RNA-Binding Properties of Bacterial Hfq. Nucleic Acids Res. 40, 1856–1867. 10.1093/nar/gkr892 22053080 PMC3287200

[B114] Sonnleitner E. Blasi U. (2014). Regulation of Hfq by the RNA Crcz in *Pseudomonas aeruginosa* Carbon Catabolite Repression. PloS Genet. 10, e1004440. 10.1371/journal.pgen.1004440 24945892 PMC4063720

[B115] Sonnleitner E. Hagens S. Rosenau F. Wilhelm S. Habel A. Jager K. E.. (2003). Reduced Virulence of a Hfq Mutant of *Pseudomonas aeruginosa* O1. Microb. Pathog. 35, 217–228. 10.1016/S0882-4010(03)00149-9 14521880

[B116] Sonnleitner E. Prindl K. Blasi U. (2017). The *Pseudomonas aeruginosa* Crcz RNA Interferes With Hfq-Mediated Riboregulation. PloS One 12, e0180887. 10.1371/journal.pone.0180887 28686727 PMC5501646

[B117] Sonnleitner E. Schuster M. Sorger-Domenigg T. Greenberg E. P. Blasi U. (2006). Hfq-Dependent Alterations of the Transcriptome Profile and Effects on Quorum Sensing in *Pseudomonas aeruginosa* . Mol. Microbiol. 59, 1542–1558. 10.1111/j.1365-2958.2006.05032.x 16468994

[B118] Soper T. Mandin P. Majdalani N. Gottesman S. Woodson S. A. (2010). Positive Regulation by Small RNAs and the Role of Hfq. Proc. Natl. Acad. Sci. U. S. A. 107, 9602–9607. 10.1073/pnas.1004435107 20457943 PMC2906882

[B119] Soper T. J. Woodson S. A. (2008). The Rpos mRNA Leader Recruits Hfq to Facilitate Annealing With Dsra sRNA. RNA 14, 1907–1917. 10.1261/rna.1110608 18658123 PMC2525945

[B120] Sousa S. A. Ramos C. G. Moreira L. M. Leitão J. H. (2010). The Hfq Gene Is Required for Stress Resistance and Full Virulence of Burkholderia Cepacia to the Nematode Caenorhabditis Elegans. Microbiology 156, 896–908. 10.1099/mic.0.035139-0 19942656

[B121] Storz G. Vogel J. Wassarman K. M. (2011). Regulation by Small RNAs in Bacteria: Expanding Frontiers. Mol. Cell 43, 880–891. 10.1016/j.molcel.2011.08.022 21925377 PMC3176440

[B122] Updegrove T. B. Zhang A. Storz G. (2016). Hfq: The Flexible RNA Matchmaker. Curr. Opin. Microbiol. 30, 133–138. 10.1016/j.mib.2016.02.003 26907610 PMC4821791

[B123] Vakulskas C. A. Potts A. H. Babitzke P. Ahmer B. M. Romeo T. (2015). Regulation of Bacterial Virulence by Csr (Rsm) Systems. Microbiol. Mol. Biol. Rev. 79, 193–224. 10.1128/MMBR.00052-14 25833324 PMC4394879

[B124] Van Puyvelde S. Steenackers H. P. Vanderleyden J. (2013). Small RNAs Regulating Biofilm Formation and Outer Membrane Homeostasis. RNA Biol. 10, 185–191. 10.4161/rna.23341 23324602 PMC3594277

[B125] Vogel J. Papenfort K. (2006). Small Non-Coding RNAs and the Bacterial Outer Membrane. Curr. Opin. Microbiol. 9, 605–611. 10.1016/j.mib.2006.10.006 17055775

[B126] Vogt S. L. Raivio T. L. (2014). Hfq Reduces Envelope Stress by Controlling Expression of Envelope-Localized Proteins and Protein Complexes in Enteropathogenic *Escherichia coli* . Mol. Microbiol. 92, 681–697. 10.1111/mmi.12581 24628810

[B127] Weilbacher T. Suzuki K. Dubey A. K. Wang X. Gudapaty S. Morozov I.. (2003). A Novel sRNA Component of the Carbon Storage Regulatory System of *Escherichia coli* . Mol. Microbiol. 48, 657–670. 10.1046/j.1365-2958.2003.03459.x 12694612

[B128] Westermann A. J. Venturini E. Sellin M. E. Forstner K. U. Hardt W. D. Vogel J. (2019). The Major RNA-Binding Protein ProQ Impacts Virulence Gene Expression in *Salmonella enterica* Serovar Typhimurium. mBio 10, e02504-18. 10.1128/mBio.02504-18 30602583 PMC6315103

[B129] Wilderman P. J. Sowa N. A. Fitz Gerald D. J. Fitz Gerald P. C. Gottesman S. Ochsner U. A.. (2004). Identification of Tandem Duplicate Regulatory Small RNAs in *Pseudomonas aeruginosa* Involved in Iron Homeostasis. Proc. Natl. Acad. Sci. U. S. A. 101, 9792–9797. 10.1073/pnas.0403423101 15210934 PMC470753

[B130] Yang J. Wang H. H. Lu Y. Yi L. X. Deng Y. Lv L.. (2021). A ProQ/FinO Family Protein Involved in Plasmid Copy Number Control Favours Fitness of Bacteria Carrying Mcr-1-Bearing IncI2 Plasmids. Nucleic Acids Res. 49, 3981–3996. 10.1093/nar/gkab149 33721023 PMC8053102

[B131] Zhang A. Schu D. J. Tjaden B. C. Storz G. Gottesman S. (2013). Mutations in Interaction Surfaces Differentially Impact E. Coli Hfq Association With Small RNAs and Their mRNA Targets. J. Mol. Biol. 425, 3678–3697. 10.1016/j.jmb.2013.01.006 23318956 PMC3640674

[B132] Zhao X. Koestler B. J. Waters C. M. Hammer B. K. (2013). Post-Transcriptional Activation of a Diguanylate Cyclase by Quorum Sensing Small RNAs Promotes Biofilm Formation in *Vibrio cholerae* . Mol. Microbiol. 89, 989–1002. 10.1111/mmi.12325 23841714 PMC3807870

[B133] Zheng A. Panja S. Woodson S. A. (2016). Arginine Patch Predicts the RNA Annealing Activity of Hfq From Gram-Negative and Gram-Positive Bacteria. J. Mol. Biol. 428, 2259–2264. 10.1016/j.jmb.2016.03.027 27049793 PMC4884477

